# DFT electronic structure investigation of chromium ion-implanted cupric oxide thin films dedicated for photovoltaic absorber layers

**DOI:** 10.1038/s41598-024-70442-2

**Published:** 2024-08-27

**Authors:** Katarzyna Ungeheuer, Konstanty W. Marszalek, Waldemar Tokarz, Marcin Perzanowski, Zbigniew Kąkol, Marta Marszalek

**Affiliations:** 1 Faculty of Computer Science, Electronics and Telecommunications, AGH University of Krakow, 30 Mickiewicza Ave., 30-059 Krakow, Poland; 2 Faculty of Physics and Applied Computer Science, AGH University of Krakow, 30 Mickiewicza Ave., 30-059 Krakow, Poland; 3 Henryk Niewodniczanski Institute of Nuclear Physics, 152 Walerego Eljasza Radzikowskiego Str., 31-342 Krakow, Poland

**Keywords:** DFT, Ion implantation, Thin films, Electronic structure, Copper oxide CuO, Electronic structure, Solar cells, Two-dimensional materials

## Abstract

This study explores the enhancement of cupric oxide (CuO) thin films for photovoltaic applications through chromium doping and subsequent annealing. Thin films of CuO were deposited on silicon and glass substrates using reactive magnetron sputtering. Chromium was introduced via ion implantation, and samples were annealed to restore the crystal structure. The optical and structural properties of the films were characterized using X-ray diffraction, spectrophotometry, and spectroscopic ellipsometry. Results indicated that implantation reduced the absorbance and conductivity of the films, while annealing effectively restored these properties. Sample implanted with 10 keV energy and 1 × 10^14^ cm^−2^ dose of Cr ions, after annealing had sheet resistance of 1.1 × 10^6^ Ω/sq compared to 1.7 × 10^6^ Ω/sq for non implanted and annealed CuO. Study of crystalline structure confirmed the importance of annealing as it reduced the stress present in the material after deposition and implantation. Density Functional Theory (DFT) calculations were performed to investigate the electronic structure and optical properties of CuO with varying levels of chromium doping. Calculations revealed an energy gap of 1.8 eV for undoped CuO, with significant changes in optical absorption for doped samples. Energy band gap determined using absorbance measurement and Tauc plot method had value of 1.10 eV for as deposited CuO. Samples after implantation and annealing had energy band gap value increased to about 1.20 eV. The study demonstrates that chromium doping and subsequent annealing can enhance the optical and electronic properties of CuO thin films, making them more efficient for photovoltaic applications.

## Introduction

The use of thin-film systems enables the reduction of raw material consumption and the weight of photovoltaic cells, which is advantageous for space applications^1^ or the creation of lightweight solar modules^2^. Developing all-oxide cells allows for utilization of non-toxic and stable materials^3^. Cupric oxide (CuO), an intrinsic p-type semiconductor, emerges as a promising candidate for the absorber layer in thin film solar cells^4^, with most commonly ZnO and TiO_2_ used as n-type window layer^5–8^. Cupric oxide is also investigated for sensing^9–12^ and photocatalytic^13,14^ applications, with doping being a common method for enhancing its properties^12,15–17^. CuO-based thin-film solar cells are expected to achieve energy conversion efficiencies of up to 30%, based on the Shockley-Quisser limit^18^ and the energy band gap of the oxide, which ranges from 1 to 1.9 eV^19–22^. However, current solar cells with copper oxide as an absorber struggle to surpass 10% efficiency^23^. Therefore, modifications are essential to enhance the properties of copper oxide thin films and improve their performance in photovoltaic and other applications.

In this study, chromium is used as a dopant, introduced via ion implantation, with sample annealing conducted to restore the crystal structure. Ion implantation method is a well known and robustly studied technique, with reliable software for modelling of this process^24–27^. It gives possibility to introduce the dopant very precisely regarding both the amount of the introduced species as well as the depth of doping. Previously we studied the chromium implantation of copper oxides, here we investigate how annealing influences the properties of implanted CuO thin films, which has never been done before^28–31^. The optical and structural characteristics of the thin films are characterized with X-ray diffraction, spectrophotometry, and spectroscopic ellipsometry measurements.

In advanced solid materials, which are of great technological interest, an understanding of properties on the atomic scale is very essential. Currently the most successful method for studying the electronic structure of matter is the Density Functional Theory (DFT)^32,33^. DFT is effectively exploit in solid state physics, physical chemistry, modern electronics, but also in metallurgy, geoscience and biochemistry, and other fields where the understanding of materials properties on the atomic scale is a necessity. It works for atoms, molecules, solids, liquids and plasmas. DFT became the tool of choice for the vast majority of researchers also due to an accessibility of commercial DFT software. One of the most popular and one of the most accurate DFT codes is WIEN2K software package^34^.

The aim of this study is to characterize how physical properties of Cr^+^ implanted CuO thin films changed after annealing, as well as to assess the information about Cr doping of CuO determined with DFT calculations in comparison to real material.

## Methods

Thin films of CuO were deposited using reactive magnetron sputtering on silicon and glass substrates. Before sputtering, the substrates were cleaned with soap and warm water, rinsed with isopropyl alcohol and immersed in alcohol for 20 min of ultrasonic bath, and finally dried with N_2_. A 99.95% purity copper target was used during the sputtering. The power and discharge current were 50 W and 80–130 mA, respectively. Before deposition, a presputtering process was carried out in Ar atmosphere. Deposition process was carried out with working gas of 100% O_2_ with pressure equal to 1.5 × 10^–2^ mbar. The substrates were heated to 150 °C during the process. The deposition time was chosen as to reach thickness of 130 nm. Ion implantation was performed at the Henryk Niewodniczanski Institute of Nuclear Physics, Polish Academy of Sciences in Krakow with doses 1 × 10^14^ cm^−2^, 5 × 10^14^ cm^−2^, 1 × 10^15^ cm^−2^, and 5 × 10^16^ cm^−2^ and energy 10 keV and 15 keV as described in work of Ungeheuer et al^29^. Chosen samples were annealed in air for 6 h at 400 °C temperature. Used energy and doses of ions should result with concentration of Cr atoms in the material as presented in Table 1. These calculations were based on results of Stopping and Range of Ions in Matter^24^ software simulations. The results say what is the Cr atomic concentration at the first 5, 10, and 30 nm in depth of the films, and in the whole film of 130 nm thickness. The highest considered dose corresponds to one Cr atom per 28 Cu and O atoms combined.

**Table 1:** Atomic concentration of Cr at different depths and for the whole thickness of 130 nm film. Calculations are based on SRIM simulations results.

Thickness [nm]	at. Cr [%], implantation parameters			
	10 keV	10 keV	10 keV	15 keV
	1 × 10^14^ cm^−2^	5 × 10^14^ cm^−2^	1 × 10^15^ cm^−2^	5 × 10^16^ cm^−2^
5	0.06	0.28	0.56	14.07
10	0.07	0.36	0.72	27.35
30	0.03	0.17	0.34	3.57
130	0.01	0.04	0.08	3.80

Structural properties of deposited oxide were studied with X-ray diffraction (XRD) using PANalytical X’Pert PRO diffractometer (Malvern Panalytical, Malvern, UK) with Cu anode (0.154 nm radiation wavelength). The measurement step was 0.05° with time per step of 8000 s. Map measurements of spectroscopic ellipsometry (SE) were performed with J.A. Woollam M 2000 ellipsometer at angle of 70° for 11 × 11 data points with a step of 0.5 mm, where the spot size is around 100 μm. Ellipsometric data analysis was performed with dedicated CompleteEASE software. Spectrophotometry measurements of absorbance were performed with AvaLight-DH-S-BAL source and AvaSpec-ULS-RS-TEC detector in transmission mode. Sheet resistance was measured with RM3000 + Test Unit from Jandel employing 4-point probe method. Optical and electrical properties were measured for films deposited on glass, while XRD measurements were done for films deposited on silicon.

In our work we have carried out calculations with full potential WIEN2K code, in the Generalized Gradient Approximation^35^. The WIEN2K software was utilized for calculations of electron structure in the ground state (at 0 K). It allows us to determine the density of states (DOS), energy gap and electromagnetic radiation absorption.

**Figure 1:**
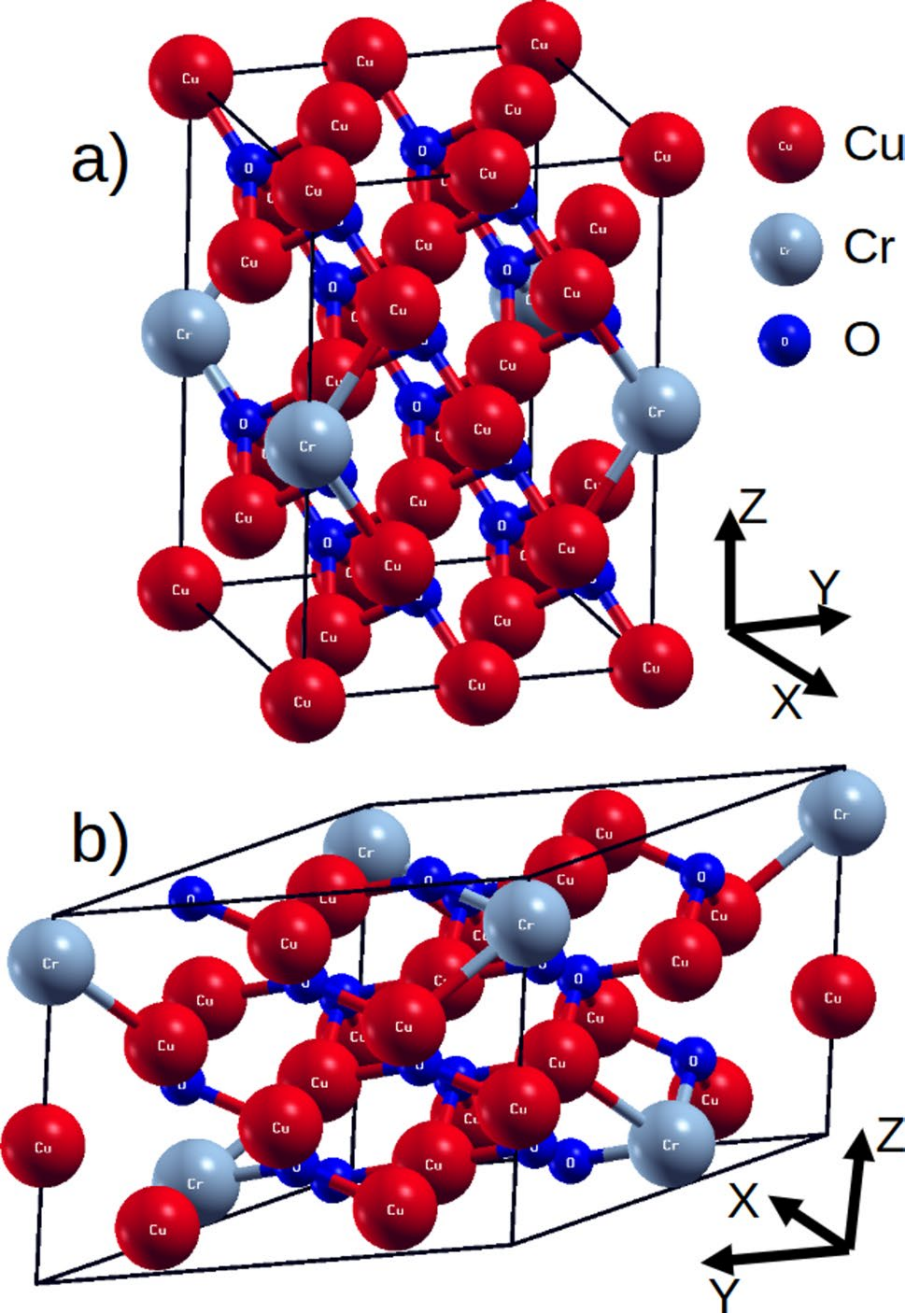
Crystal structure used in the calculation (**a**) Cu_7_CrO_8_ and (**b**) Cu_15_CrO_16_.

As a starting point we utilized CuO structure C2/c (ITC 15) with lattice parameters *a* = 4.66 Å, *b* = 3.49 Å, *c* = 5.13 Å, *α*, *γ* = 90°, *β* = 98.71°. This structure was extended 2 × 2 × 2 times in order to incorporate proper initial antiferromagnetic spin arrangement^36^ on Cu sites. Second considered structure was with one chromium substituted in place of Cu Cu_15_CrO_18_. After symmetry consideration this structure was reduced to P-1 (ITC 2) with *a* = 5.11 Å, *b* = 6.82 Å, *c* = 9.31 Å *α*, *γ* = 90°, *β* = 99.48° (Fig. 1 a). Third system was constructed by arbitrarily choosing two Cu atoms and replacing them with Cr, as result Pc (7 ITC) structure with lattice parameters *a* = 9.85 Å, *b* = 9.31 Å, *c* = 6.82 Å *α*, *β* = 90°, *γ* = 149.24° was obtained (presented on Fig. 1b). We theoretically calculated the electronic properties for two different Cr concentrations to illustrate the direction of changes that occur in the material as it is doped. Additional for Cu and Cr d states orbital dependent potentials LDA + U(SIC)^37^ with effective potential 0.52 Ry was used. The second system has Cr concentration similar to the atomic Cr concentration in sample implanted with dose 5 × 10^16^ cm^−2^ and energy 15 keV.

## Results

**Figure 2:**
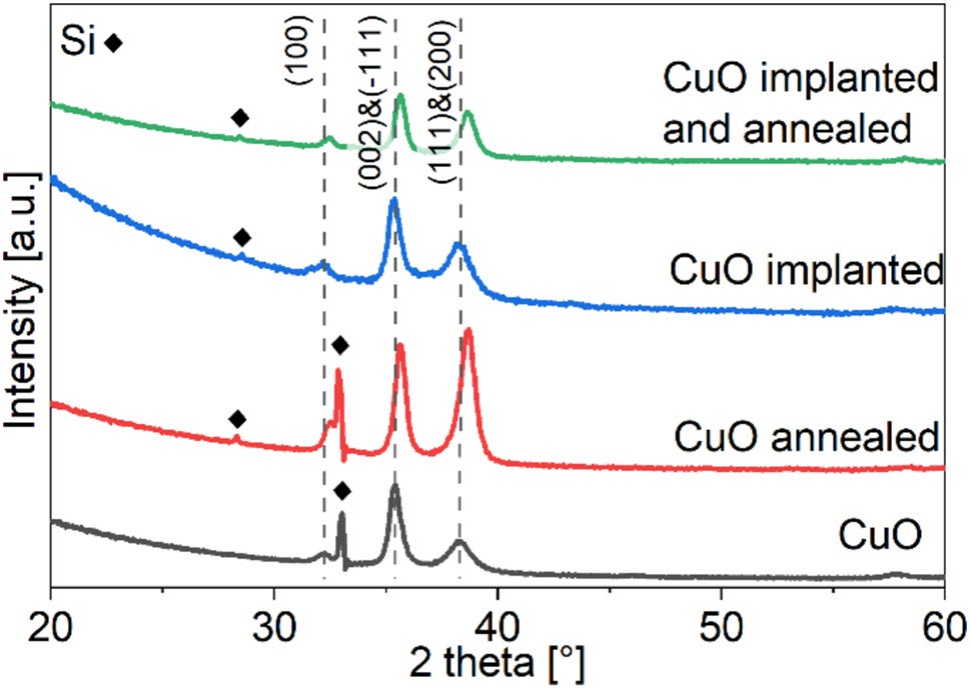
XRD data with the most intensive CuO peaks visible, peaks coming from silicon substrate are designated with a marker, implantation of these samples was done at 15 keV energy and 5 × 10^16^ cm^−2^ dose of Cr^+^ ions.

XRD data for four samples of CuO is presented in Fig. 2, the samples are: film after deposition, sample after annealing, implanted sample, and an implanted and annealed sample. We designated the crystalline peaks of CuO basing on ICDD card #01-080-0076. The annealing process caused a shift of the peaks to the higher angle values. Shift to higher angle means a decrease of interplanar distances, their values were used to calculate the lattice parameters of CuO cell. Shift of peaks position can be caused in by residual stress or stress induced by the implantation^38,39^. We calculated the lattice parameters of CuO using the positions of peaks (− 111), (100) and (− 311), the last one is not visible in Fig. 2 to assure the clearance of the data presentation and avoid strong Si peak around 70°.

The calculation of *a*, *b*, *c* and *β* parameters of CuO monoclinic crystal structure follows Eqs. (1) to (5), Eq. 1 is the equation for interplanar distance calculation for monoclinic structure^40^, and other Equations are calculated for h, k, l values of (− 111), (− 311) and (110) peaks.

1 $$\frac{1}{{d}_{hkl}^{2}}=\frac{{h}^{2}}{{a}^{2}{\text{sin}}^{2}\beta }+\frac{{k}^{2}}{{b}^{2}}+\frac{{l}^{2}}{{c}^{2}{\text{sin}}^{2}\beta }-\frac{2hl\text{cos}\beta }{ac{\text{sin}}^{2}\beta }$$

2 $$\text{cos}\beta =\frac{\sqrt{2}}{2}\left(\frac{1}{{d}_{-111}^{2}}-\frac{1}{{d}_{111}^{2}}\right)\frac{1}{\sqrt{\frac{1}{{d}_{-311}^{2}}-\frac{2}{{d}_{-111}^{2}}+\frac{1}{{d}_{111}^{2}}}}\frac{1}{\sqrt{\frac{1}{{2d}_{-111}^{2}}-\frac{1}{{d}_{110}^{2}}+\frac{1}{{2d}_{111}^{2}}}}$$

3 $$a=\frac{2\sqrt{2}}{sin\beta }\frac{1}{\sqrt{\frac{1}{{d}_{-311}^{2}}-\frac{2}{{d}_{-111}^{2}}+\frac{1}{{d}_{111}^{2}}}}$$

4 $$c=\frac{1}{sin\beta }\frac{1}{\sqrt{\frac{1}{{2d}_{-111}^{2}}-\frac{1}{{d}_{110}^{2}}+\frac{1}{{2d}_{111}^{2}}}}$$

5 $$b=\frac{asin\beta {d}_{110}}{\sqrt{{a}^{2}{\text{sin}}^{2}\beta -{d}_{110}^{2}}}$$

Results of lattice parameters calculation are presented in Table 2. The implantation increased the value of *β* angle, which after annealing returns to a lower value. The parameter *a* has no noticeable change either with implantation nor annealing, two other parameters, *b* and *c*, both get smaller after implantation, the structure is compressed. Annealing of non-implanted sample reduced *a* and *c* parameters, and increased *b* and *β.* In case of implanted sample annealing caused decrease of *c* parameter and the *β* angle. A clear effect as seen in Fig. 2 is that annealing changes the ratio of (002) & (− 111) to (111) & (200) peaks intensity, which can be observed for both implanted and nonimplanted samples.

**Table 2:** Lattice parameters of CuO monoclinic structure for films deposited on silicon, and implanted and/or annealed.

Sample	*a* [Å]	*b* [Å]	*c* [Å]	*β* [°]
CuO	4.67	3.47	5.15	98.61
CuO annealed	4.66	3.49	5.13	98.71
CuO implanted 15 keV	4.68	3.42	5.12	99.02
CuO implanted 15 keV annealed	4.69	3.43	5.10	98.91

**Figure 3:**
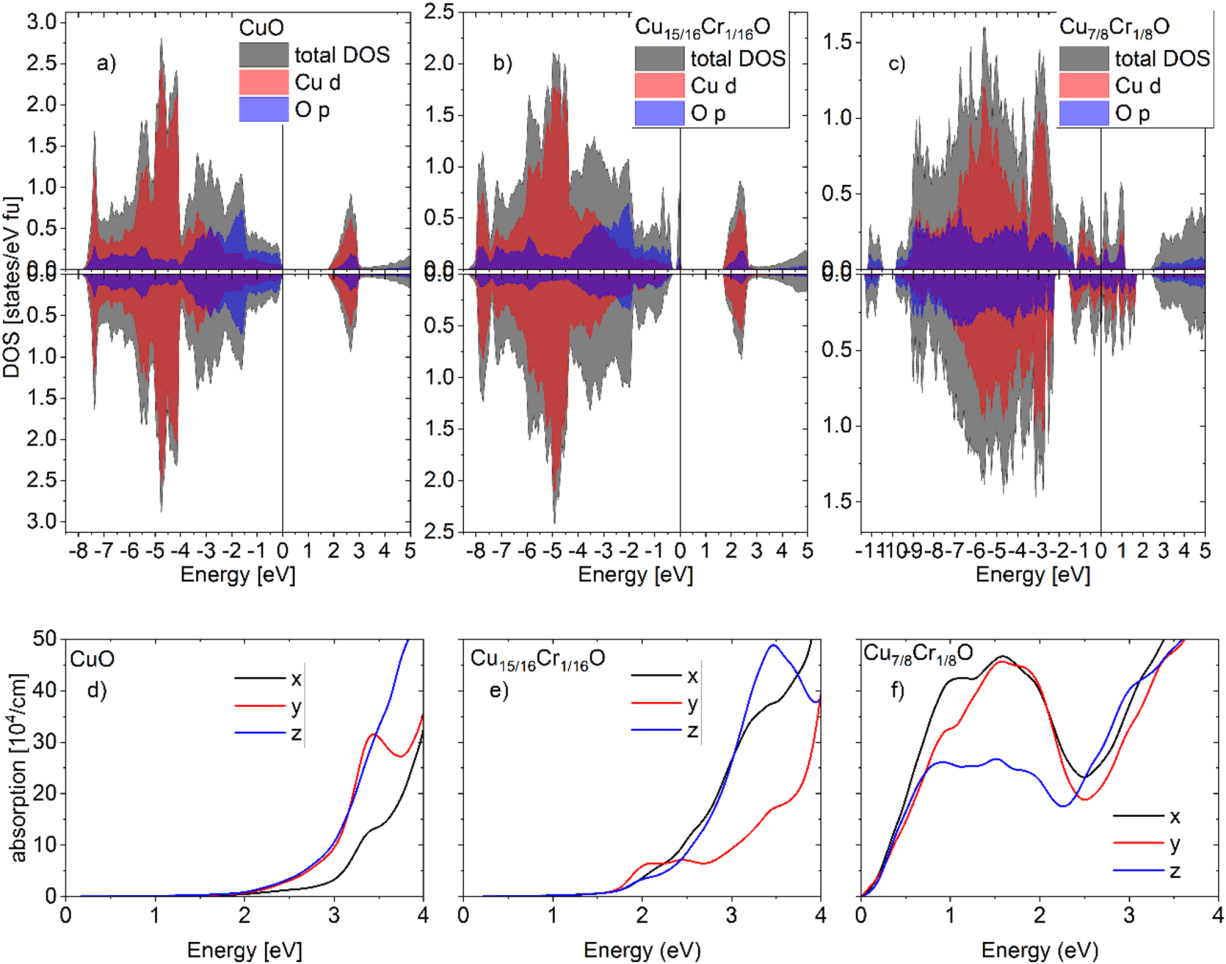
Calculated density of states (**a**, **b**, **c**) and absorption of electromagnetic wave (**d**, **e**, **f**) for CuO (**a**, **d**) Cu_15_CrO_16_ (**b**, **e**) and Cu_7_CrO_8_ (**c**, **f**). The x, y and z directions are defined in Fig. 1.

From the calculation of the electron structure, we obtained the Density of States (DOS) with an energy gap of 1.75 eV (Fig. 3a) for CuO, consistent with the experimentally measured values^41,42^ Note that this calculated value corresponds to ground state (T = 0 K) and that band-gap energy of semiconductors tends to decrease with increasing temperature as in Eq. (6)

6 $$E_{g} = E_{0} {-}\alpha T^{{2}} /\left( {T \, + \, \beta } \right)$$

where *α* and *β* are constants^43^. In the case of Cu_15_CrO_16_, at the Fermi energy level there is a very narrow half metallic conduction band for electrons with spin up and an energy gap of 1.6 eV for the spin up and 1.9 eV for the spin down (see Fig. 3b). Doping at the level of 1 Cr atom per 7 Cu atoms, we have a metallic conduction band (Fig. 3c). In all considered cases, the valence band is mainly formed by d-type Cu and p-type O electrons (Fig. 3a, b and c).

Optical absorption presented in the Fig. 3d, e and f was calculated in Wien2k package based on a scheme for the calculation of linear optical properties by the all-electron full-potential linearized augmented planewave (LAPW) method^44^. In CuO, absorption begins for photons with energy above 1.7 eV (Fig. 3d) and for the Cr dopant at the level of one atom per 15 copper atoms, it increases significantly from 1.6 eV Fig. 3e), while for Cu_7_CrO_**8**_ it occurs from the lowest energies (Fig. 3f).

Measurement of absorbance is possible only up to 3.5 eV energy of light, as the glass substrate starts absorbing above that energy.

To estimate the band gap *E*_*g*_ of material, we use the Tauc’s plot method. For CuO with indirect allowed transition^23,45–47^ the plot is created as in Eq. (7)^48,49^.

7 $${\left(\alpha h\nu \right)}^\frac{1}{2}=B\left(h\nu -{E}_{g}\right)$$

**Figure 4:**
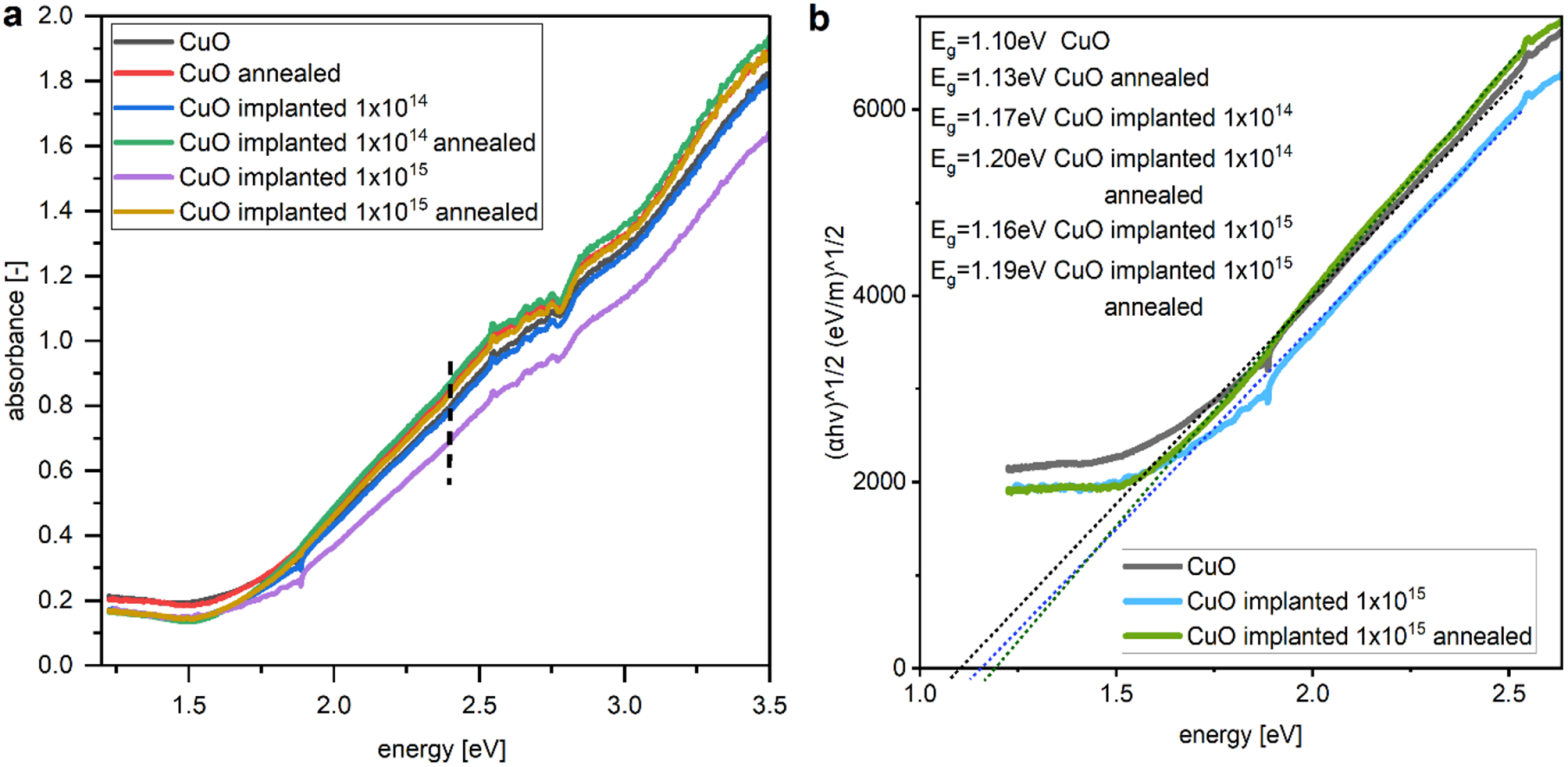
(**a**) Absorbance of CuO films after different processes, a dashed line marks the light energy used for establishing the pseudo refractive index parameter in SE maps, (**b**) Tauc’s plot example for three chosen samples with calculated optical energy band gaps for all considered samples.

All the films were deposited with the same parameters and deposition time thus we assume they have the same thickness, which enables to compare the absorbance of the samples. The implantation reduces absorbance of CuO thin films, with stronger effect for higher dose of ions (Fig. 4a). Annealing step restores absorbing properties of the oxide, which are crucial in photovoltaic applications, with resulting higher absorbance for implanted and annealed samples than only annealed one. Though the absorbance rises after annealing, so does the energy band gap estimated using Tauc’s plot method (Fig. 4b). The largest energy band gap was calculated for sample implanted with Cr + ions of 10 keV energy and 1 × 10^14^ cm^−2^ dose and then annealed in air. The measured optical absorption is with accordance to results obtained from DFT calculation for CuO and Cu_7_CrO_8_ (Fig. 3d, e).

**Figure 5:**
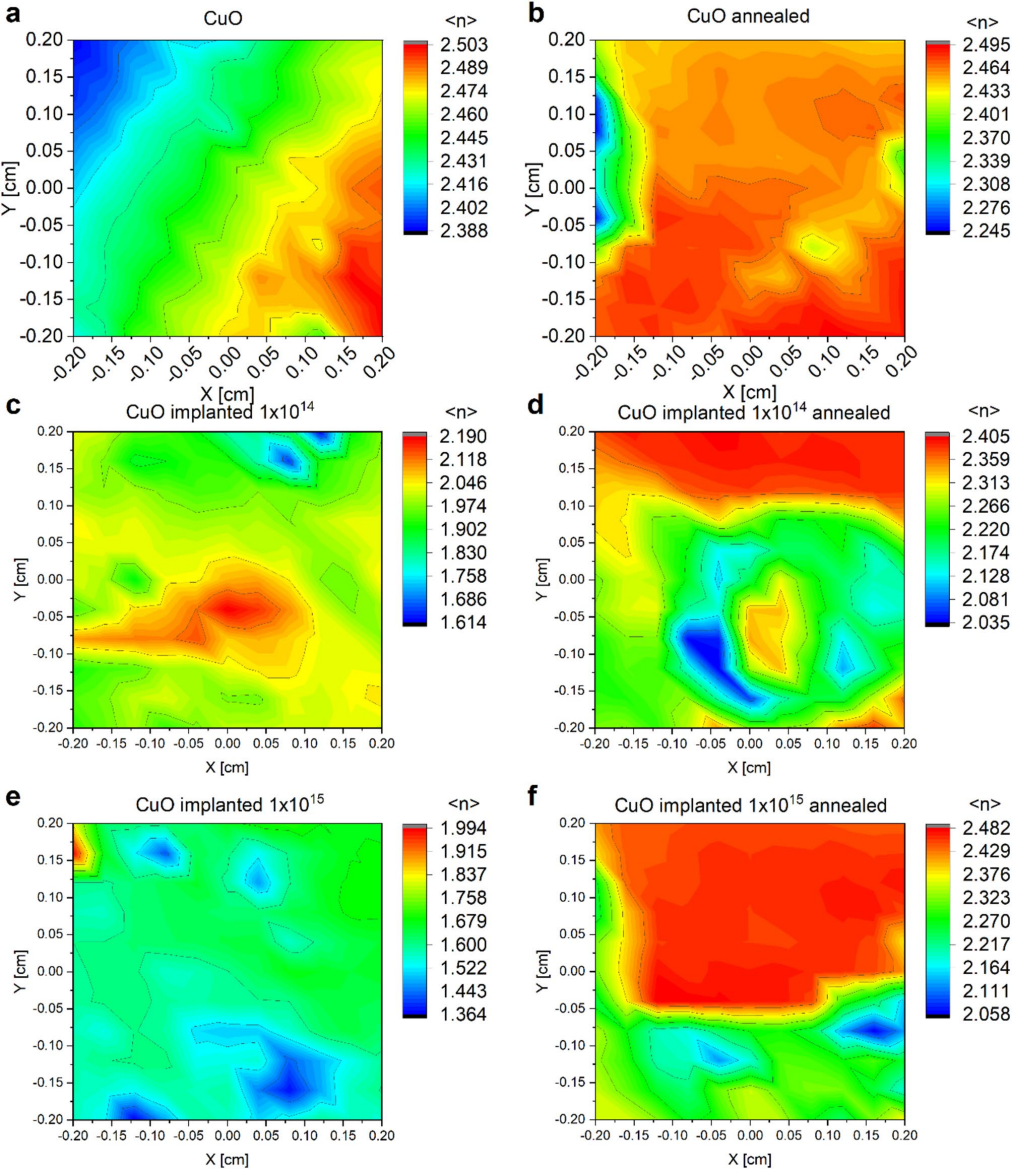
Maps of pseudo refractive index < *n* > for CuO thin films (**a**) after deposition, (**b**) after annealing, (**c**) implanted with Cr^+^ ions of 10 keV energy and 1 × 10^14^ cm^−2^ dose, (**d**) and then annealed, (**e**) implanted with Cr^+^ ions of 10 keV energy and 1 × 10^15^ cm^−2^ dose, (**f**) and then annealed.

Spectroscopic ellipsometry was used to study the homogeneity of thin films. The parameter presented in Fig. 5 is pseudo refractive index < *n* > defined by Eqs. 8 and 9^50,51^. It should not be regarded solely as an optical property of the deposited layers or the substrate; rather, it represents the refractive index of the entire sample. For bulk samples, it signifies the optical property of the material under consideration. More importantly this parameter depends on both values that are measured in the SE method–Psi *Ψ* and Delta *Δ*.

8 $${\langle \widetilde{n}\rangle }^{2}={\left(\langle n\rangle +i\langle k\rangle \right)}^{2}={\text{sin}\varphi }^{2}\cdot \left[1+{\text{tan}\varphi }^{2}\cdot {\left(\frac{1-\rho }{1+\rho }\right)}^{2}\right]$$

where

9 $$\rho =\text{tan}\Psi \cdot {e}^{i\Delta }$$

$\widetilde{n}$– complex refractive index, *n* – refractive index, *k*—extinction coefficient, *φ* – angle of incidence, *Ψ* – amplitude ratio, and *Δ* – phase difference ratio of polarized light.

Using this parameter allows to skip the process of preparing a complicated model of a sample, and to quickly asses the changes of optical parameters, as well as roughness of thin films. It is clear from Fig. 5a that there is a slope of thickness on as deposited CuO thin film, which is caused by lack of rotation during the deposition with magnetron sputtering method. Samples after implantation do not show this slope, but they have some spots of nonhomogeneity (Fig. 5c, d). After the annealing, the value of pseudo refractive index increases for implanted samples, and the films look more homogenous (Fig. 5d, f).

Sheet resistance measurements results are presented in Table 3. Annealing of pristine CuO film reduced the electrical conductivity, while for implanted samples, the annealing process resulted with improvement of conductivity.

**Table 3:** Sheet resistance of CuO thin films measured with 4 point probe method.

Sample	Sheet resistance [Ω/sq] at 100 nA
CuO	8.2 × 10^5^ ± 5.3 × 10^2^
CuO annealed	1.7 × 10^6^ ± 3.6 × 10^2^
CuO implanted dose 1 × 10^14^ cm^−2^	8.0 × 10^6^ ± 2.0 × 10^3^
CuO implanted dose 1 × 10^15^ cm^−2^	–
CuO implanted dose 1 × 10^14^ cm^−2^ annealed	1.1 × 10^6^ ± 6.5 × 10^2^
CuO implanted dose 1 × 10^15^ cm^−2^ annealed	6.3 × 10^6^ ± 7.7 × 10^3^

## Conclusions

DFT method yields electronic structure and allows computing of variety materials parameters.

Key findings from these calculations include:

- An energy gap *E*_*g*_ of 1.8 eV was found for CuO. This value is consistent with the experimentally measured values, though the value measured using spectrophotometry is much lower than calculated (1.10 eV), this difference is due to the fact that DTF calculations consider the ground state, while the optical properties were measured at room temperature. Moreover, the calculations give information about a perfect crystal, while the samples are polycrystalline, they have grain boundaries, point defects and impurities;
- In Cu_15_CrO_16_, we observe a very narrow half metallic conduction band at the Fermi energy level for electrons with spin up and with an energy gap of 1.6 eV for the spin up and 1.9 eV for the spin down;
- Doping at the level of 1 Cr atom per 7 Cu atoms, gives a metallic conduction band, four-point probe measurements of sheet resistance show that doped films still have high resistance and do not behave as metallic material;
- In all considered cases, the valence band is mainly formed by d-type Cu and p-type O electrons;
- Optical absorption in CuO begins for the photons energy above 1.8 eV;
- With Cr doping level of one atom per 15 copper atoms, optical absorption increases significantly starting of 1.6 eV, while for Cu_7_CrO_**8**_ it rises from the lowest energies.

We studied how Cr^+^ ion implantation and further annealing in air influences the properties of CuO. We can say that implantation reduces both absorbance and conductivity of the films, while annealing enables to restore these two properties. Tough the optical energy band gap estimated using Tauc’s plot method shows that used modifications increase the band gap of CuO from 1.10 eV to about 1.20 eV for implanted and annealed samples. CuO thin films are to be used as absorbing layer in photovoltaic cells, the homogeneity of the film is crucial. The changes of homogeneity of the optical and physical properties over the surface of the sample were determined using pseudo refractive index parameter. The annealing process improved the homogeneity of the films, both after deposition and implantation. The annealing step is crucial after implantation to relax the stress in the structure of the material and to homogenize the distribution of the implanted species.

## Data Availability

Data is available on request from the corresponding author.
